# Predicting clinically significant prostate cancer with or without digital rectal exam and MRI data using ClarityDX Prostate models

**DOI:** 10.1038/s41746-026-02642-1

**Published:** 2026-04-17

**Authors:** Robert J. Paproski, Adam Kinnaird, M. Eric Hyndman, Adrian Fairey, Leonard Marks, Christian P. Pavlovich, Sean A. Fletcher, Roman Zachoval, Vanda Adamcova, Jiri Stejskal, Armen Aprikian, Christopher J. D. Wallis, Desmond Pink, Catalina Vasquez, Perrin H. Beatty, John D. Lewis

**Affiliations:** 1 Nanostics Inc., Edmonton, AB Canada; 2 Division of Urology, Department of Surgery, University of Alberta, Kipnes Urology Centre, Edmonton, AB Canada; 3 Department of Oncology, University of Alberta, Edmonton, AB Canada; 4 Department of Surgical Oncology, University of Calgary, Prostate Cancer Centre, Calgary, AB Canada; 5 UCLA Health, Los Angeles, CA USA; 6 James Buchanan Brady Urological Institute, Johns Hopkins University School of Medicine, Baltimore, MD USA; 7 Department of Urology, 3rd Faculty of Medicine of Charles University and Thomayer University Hospital, Prague, Czech Republic; 8 Department of Surgery, McGill University, Montreal, QC Canada; 9 Division of Urology, Department of Surgery, University of Toronto, Toronto, ON Canada; 10 Division of Urology, Department of Surgery, Mount Sinai Hospital, Toronto, ON Canada

**Keywords:** Health care, Medical research, Risk factors, Biomarkers, Predictive markers, Cancer screening, Diseases, Prostate cancer

## Abstract

This prognostic study created optimized ensembles of calibrated random forest models to predict clinically significant prostate cancer (csPCa, grade group ≥2 PCa) using total prostate-specific antigen (PSA), free PSA, negative biopsy status, and age, with or without DRE and MRI data. Observational data were aggregated from cohorts in six organizations in Canada, the USA, and Czechia. Prostate biopsies were performed between 2009 and 2024. Risk models (ClarityDX Prostate + DRE, ClarityDX Prostate + MRI, and ClarityDX Prostate + MRI + DRE) were derived (training cohorts *n* = 1626 to 2191) and validated (validation cohorts *n* = 378 to 1318) from different clinical sites. The models had ROC AUC values ≥ 0.80. Adding DRE improved the ROC AUC to 0.82 while models using MRI features had ROC AUC values of 0.87 (without DRE) and 0.88 (with DRE) in the validation cohort. These four ClarityDX Prostate models offer high accuracy in predicting csPCa in individuals in variable clinical settings.

## Introduction

Prostate cancer (PCa) is the most common solid organ cancer in men and the fifth leading cause of cancer-related deaths globally^1^. Early detection of PCa by screening with the prostate-specific antigen (PSA) blood test has been associated with decreased mortality^2^. However, over the last 20 years, recommendations regarding PSA-based PCa screening have varied as this mortality benefit is offset by a lack of specificity, contributing to significant morbidity as a result of over-investigation and overdiagnosis of clinically indolent disease^3^. Recent evidence suggests that reductions in PSA screening have led to increases in both late-stage diagnosis and PCa mortality^4^.

There is a clear unmet clinical need for early detection of clinically significant prostate cancer (csPCa; grade group (GG) ≥ 2, corresponding to a Gleason score of 3 + 4 = 7^5^) to reduce PCa mortality while minimizing patient harm from overdiagnosis of indolent disease and unnecessary prostate biopsies, which have a ~ 2% risk of hospitalization^6,7^.

The American Urological Association (AUA), European Association of Urology (EAU), and the Canadian Urological Association (CUA) now recommend men with elevated PSA use adjunctive strategies such as multiparametric MRI (mpMRI), percent-free PSA, risk calculators, or biofluid biomarker tests before biopsy to counter overdiagnosis and overtreatment^4,6^. This allows better risk stratification and identification of those men at the highest risk of harboring PCa that requires treatment.

MRI has become a common pre-biopsy procedure for lesion detection and grading with the Prostate Imaging Reporting and Data System (PI-RADS) which provides good sensitivity and negative predictive value for clinically significant disease^8,9^. Using mpMRI before prostate biopsy can reduce the number of biopsies performed by about 30% while also increasing the diagnosis of clinically significant disease^9–11^.

However, patients in many countries have limited, delayed, or no access to mpMRI due to lack of availability, lack of experienced technicians, and the high cost of the machinery^12,13^. In addition, performing DREs has been declining in clinical practice, and in family medicine residents’ training in, knowledge about, and perceptions of DREs have been negative^14^. Recent evidence shows that DRE holds little merit on its own as a diagnostic test but in combination with other clinical factors and biomarkers, DRE adds value^15,16^.

Diagnostic biomarker tests such as 4Kscore, prostate health index (PHI), and PCa risk models including ERSPC risk calculators, the PCa Prevention Trial Risk Calculator (PCPTRC), and the Prostate Biopsy Collaborative Group (PBCG) are available biomarker assays and risk calculators recognized by the AUA^6,17–21^. These tests can also help patients, and their healthcare providers make informed decisions by generating a risk score for disease^6,17–21^.

MRI-based PCa risk calculators (Mehralivand, Radtke, PLUM, Imperial RAPID, Leeuwen, and PCRC-MRI, Supplementary Table S1) have been developed as tools for early detection of csPCa, although not all of these have been validated yet^8,11,19,21–27^.

We recently developed a test called ClarityDX Prostate which predicted csPCA with a nearly 3-fold increase in specificity compared to PSA testing alone using readily available patient clinical characteristics and approved laboratory tests^28^.

To provide an accurate prediction of csPCa in a greater range of clinical settings, which may or may not have DRE and MRI data, we created models to predict csPCa with or without DRE and MRI data. The MRI data included prostate volume and the highest PI-RADS score. The ClarityDX Prostate + DRE, ClarityDX Prostate + MRI, and ClarityDX Prostate + MRI + DRE models were compared with 13 publicly available PCa risk calculators to verify their state-of-the-art accuracy.

## Results

### Clinical features

Patient characteristics for the training and validation cohorts for ClarityDX Prostate models without MRI data have been previously described^28^. For the datasets used to create and validate ClarityDX Prostate models using MRI data, the median patient age was 64 to 65 years at all four clinical sites (Table 1). At least 64% of patients were Caucasian at both UCLA, JHU, and CU (ethnicity data not available at TUH). JHU and CU had the highest proportion of African Americans at 18%. TUH and JHU had the highest and lowest percentage of patients with a prior negative biopsy at 50% and 22%, respectively. Of those patients with known DRE status, 19%, 18%, 11%, and 8% had an abnormal DRE at JHU, UCLA, CU, and TUH, respectively. Median total PSA was slightly higher at UCLA at 6.6 ng/mL compared to JHU and TUH at 6.2 ng/mL. The free PSA ratio was similar between sites at 0.15 to 0.16. At least 64% of patients from all sites had a PI-RADS score ≥3. Grade group ≥2 PCa (i.e., csPCa) was observed in 29%, 37%, 42%, and 42% of patients from CU, TUH, JHU, and UCLA, respectively. The training and validation cohorts had 42% and 35% of patients with csPCa, respectively. When comparing training cohorts for ClarityDX Prostate models without and with MRI, there were no significant differences between age, PSA, free PSA ratio, and proportion of patients with csPCa (*p* value > 0.05), suggesting that the removal of patients without MRI data had a minor impact on patient characteristics in the training cohort.

**Table 1:** Patient characteristics with pre-biopsy MRI by clinical site

	Training cohort		Validation cohort	
	University of California, Los Angeles	Johns Hopkins University	Thomayer University Hospital	ChesapeakeUrology
**Patients,** *n*	1386	240	317	61
**Age, years, median (IQR)**	65 (59–70)	65 (60–70)	64 (57–68)	65 (60–70)
**Ethnicity,** *n* **(%)**			N/A	
**African American**	71 (5.1%)	44 (18%)		11 (18%)
**Asian**	84 (6.1%)	7 (2.9%)		2 (3.3%)
**Caucasian**	928 (67%)	175 (73%)		39 (64%)
**Hispanic**	8 (0.58%)	6 (2.5%)		1 (1.6%)
**Native American**	1 (0.072%)	0 (0.0%)		0 (0.0%)
**Other**	63 (4.5%)	5 (2.1%)		8 (13%)
**Unknown**	231 (17%)	3 (1.2%)		0 (0.0%)
**Family history PCa,** *n* **(%)**		N/A	N/A	N/A
**Yes**	335 (24%)			
**No**	1012 (73%)			
**Unknown**	39 (2.8%)			
**DRE,** *n* **(% abnormal)**				
**Yes**	186 (13%)	43 (18%)	25 (7.9%)	7 (11%)
**No**	834 (60%)	184 (77%)	292 (92%)	54 (89%)
**Unknown**	366 (26%)	13 (5.4%)	0 (0.0%)	0 (0.0%)
**Prior negative biopsy,** *n* **(%)**				
**Yes**	515 (37%)	52 (22%)	160 (50%)	17 (28%)
**No**	871 (63%)	188 (78%)	157 (50%)	44 (72%)
**Unknown**	0 (0.0%)	0 (0.0%)	0 (0.0%)	0 (0.0%)
**PSA, ng/ml, median (IQR)**	6.6 (4.8–9.4)	6.2 (4.6–8.3)	6.2 (4.2–9.4)	6.3 (4.7–8.4)
**Free PSA ratio, median (IQR)**	0.15 (0.10–0.20)	0.16 (0.11–0.21)	0.15 (0.11–0.19)	0.16 (0.11–0.22)
**Prostate volume (MRI), cc, median (IQR)**	47 (35–65)	49 (36–68)	47 (35–66)	58 (39–70)
**Pre-biopsy PI-RADS,** *n* **(%)**				
**PI-RADS** ≤ **2**	350 (25%)	50 (21%)	51 (16%)	22 (36%)
**PI-RADS** ≥ **3**	1036 (75%)	190 (79%)	266 (84%)	39 (64%)
**Unknown**	0 (0.0%)	0 (0.0%)	0 (0.0%)	0 (0.0%)
**Prostate biopsy results,** *n* **(%)**				
**Negative**	558 (40%)	77 (32%)	157 (50%)	31 (51%)
**Grade group 1** **PCa**	250 (18%)	63 (26%)	44 (14%)	12 (20%)
**Grade group 2** **PCa**	265 (19%)	45 (19%)	50 (16%)	7 (11%)
**Grade group 3** **PCa**	117 (8.4%)	22 (9.2%)	26 (8.2%)	4 (6.6%)
**Grade group 4** **PCa**	84 (6.1%)	9 (3.8%)	23 (7.3%)	5 (8.2%)
**Grade group 5** **PCa**	112 (8.1%)	24 (10%)	17 (5.4%)	2 (3.3%)

Individual laboratory tests and clinical features were assessed for their ability to predict grade group ≥2 PCa in the UCLA and JHU training cohort (Table 2). Seven features including age, total PSA, free PSA ratio, prostate volume from MRI, PI-RADS score, DRE, and prior negative biopsy status were significantly different between patients with and without grade group ≥2 PCa with *p* values < 0.0001. These features were selected as inputs for ClarityDX Prostate models with DRE as optional. All ethnicities and family history were not significantly different between groups after adjusting *p* values for multiple comparisons. For individual features, the highest PI-RADS score had the highest ROC AUC at 0.77, followed by free PSA ratio (0.69), prostate volume (0.68), total PSA (0.63), age (0.61), DRE (0.58), and previous negative biopsy status (0.58).

**Table 2:** Predictive value of single features for grade group ≥2 prostate cancer in the training cohort with pre-biopsy MRI

	GG ≤ 1 PCa	GG ≥ 2 PCa	*p* value	ROC AUC (CI)
**Patients,** *n*	948 (58%)	678 (42%)		
**African American,** *n* **(%)**	57 (6.0%)	58 (8.6%)	0.35	0.51 (0.50–0.53)
**Asian,** *n* **(%)**	61 (6.4%)	30 (4.4%)	0.60	0.51 (0.50–0.52)
**Caucasian,** *n* **(%)**	654 (69%)	449 (66%)	1.00	0.51 (0.49–0.54)
**Hispanic,** *n* **(%)**	9 (0.95%)	5 (0.74%)	1.00	0.50 (0.50–0.51)
**Native American,** *n* **(%)**	0 (0.0%)	1 (0.15%)	1.00	0.50 (0.50–0.50)
**Ethnicity other,** *n* **(%)**	33 (3.5%)	35 (5.2%)	0.60	0.51 (0.50–0.52)
**Family history PCa,** *n* **(%)**	203 (21%)	132 (19%)	1.00	0.51 (0.49–0.53)
**Previous negative biopsy,** *n* **(%)**	390 (41%)	177 (26%)	**<0.0001**	0.58 (0.55–0.60)
**Age, yr, median (IQR)**	64 (59–69)	67 (61–72)	**<0.0001**	0.61 (0.58–0.63)
**DRE,** *n* **(% abnormal)**	69 (7.3%)	160 (24%)	**<0.0001**	0.58 (0.55–0.60)
**PSA, ng/ml, median (IQR)**	6.1 (4.4–8.4)	7.4 (5.4–12)	**<0.0001**	0.63 (0.60–0.66)
**Free PSA ratio, median (IQR)**	0.17 (0.13–0.22)	0.12 (0.090–0.17)	**<0.0001**	0.69 (0.66–0.71)
**Prostate volume MRI, median (IQR)**	54 (40–73)	40 (32–54)	**<0.0001**	0.68 (0.65–0.70)
**PI-RADS score (highest), median (IQR)**	3.0 (0.0–4.0)	4.0 (3.0–5.0)	**<0.0001**	0.77 (0.75–0.79)

The bold values are p values ≤0.05.

### Predictive model development and performance optimization

Four different ClarityDX Prostate models ( ± MRI ± DRE) were derived from the training cohorts using features described in Supplementary Table S1. For each clinical site, the ROC AUC for all ClarityDX Prostate models were compared to other risk calculators that use relatively similar features based on the availability of DRE and MRI data as described in Table 3. ClarityDX Prostate ROC AUC ranged from 0.793 to 0.836 for all six clinical sites with an average ROC AUC of 0.814 for the 3 validation sites of UA, TUH, and CU. ClarityDX Prostate had significantly higher ROC AUC values than ERSPC-3/4, PCPTRC, and PBCG with *p* values < 0.01 for UCLA, JHU, UC, UA, and TUH. ClarityDX Prostate + DRE ROC AUC values were at least 0.011 higher for all clinical sites compared to ClarityDX Prostate with ROC AUC values between 0.813 and 0.851. ClarityDX Prostate + DRE had significantly higher ROC AUC values than ERSPC-3/4, PCPTRC, and PBCG with *p* values < 0.01 for UCLA, JHU, UC, UA, and TUH.

**Table 3:** Risk calculator ROC AUC values with or without MRI and DRE data for predicting grade group ≥2 prostate cancer by clinical site

	ROC AUC					
	University of California, Los Angeles	Johns Hopkins University	University of Calgary	University of Alberta	Thomayer University Hospital	Chesapeake Urology
**No MRI, No DRE**						
ERSPC-3/4	N/A	0.630	0.715	0.756	0.642	0.720
PCPTRC	0.677	0.588	0.676	0.747	0.679	0.702
PBCG	0.711	0.634	0.709	0.765	0.726	0.747
ClarityDX Prostate	**0.802**	**0.793**	**0.821**	**0.804**	**0.803**	**0.836**
**No MRI, DRE available**						
ERSPC-3/4	N/A	0.699	0.725	0.772	0.683	0.727
PCPTRC	0.688	0.627	0.701	0.766	0.695	0.734
PBCG	0.713	0.686	0.733	0.785	0.741	0.779
ClarityDX Prostate + DRE	**0.813**	**0.814**	**0.833**	**0.817**	**0.815**	**0.851**
**MRI available, No DRE**						
MSP-RC	N/A	0.771	N/A	N/A	0.823	0.886
ERSPC-3/4 MRI	N/A	0.751	N/A	N/A	0.834	0.848
SPCC	N/A	0.777	N/A	N/A	0.842	**0.899**
Mehralivand	0.853	0.793	N/A	N/A	0.841	0.875
Radtke	0.844	0.786	N/A	N/A	0.843	0.864
PLUM	0.851	0.801	N/A	N/A	0.849	0.895
Imperial RAPID	0.849	0.793	N/A	N/A	0.849	0.891
BCN2RC	N/A	0.797	N/A	N/A	0.844	0.854
Leeuwen	0.834	0.779	N/A	N/A	0.853	0.833
PCRC-MRI	0.855	0.798	N/A	N/A	0.856	0.881
ClarityDX Prostate +MRI	**0.892**	**0.887**	N/A	N/A	**0.871**	0.893
**MRI available, DRE available**						
MSP-RC	N/A	0.771	N/A	N/A	0.823	0.886
ERSPC-3/4 MRI	N/A	0.768	N/A	N/A	0.835	0.846
SPCC	N/A	0.777	N/A	N/A	0.842	**0.899**
Mehralivand	0.856	0.800	N/A	N/A	0.842	0.875
Radtke	0.850	0.798	N/A	N/A	0.846	0.853
PLUM	0.851	0.801	N/A	N/A	0.849	0.895
Imperial RAPID	0.849	0.793	N/A	N/A	0.849	0.891
BCN2RC	N/A	0.806	N/A	N/A	0.849	0.851
Leeuwen	0.836	0.793	N/A	N/A	0.854	0.832
PCRC-MRI	0.858	0.810	N/A	N/A	0.857	0.877
ClarityDX Prostate + MRI + DRE	**0.895**	**0.890**	N/A	N/A	**0.874**	0.894

Highest ROC AUC values bolded in each DRE/MRI category.

Adding MRI features significantly increased model performance since ClarityDX Prostate + MRI had ROC AUC values at least 0.057 higher than the ClarityDX Prostate model (*p* value < 0.005 for the validation cohort including TUH and CU). ClarityDX Prostate + MRI had ROC AUC values at least 0.015, 0.037, and 0.086 greater than all 10 MRI-based risk calculators for TUH, UCLA, and JHU, respectively. Similar results were observed when MRI and DRE results were available since ClarityDX Prostate + MRI + DRE had ROC AUC values at least 0.017, 0.037, and 0.080 greater than all 10 MRI-based risk calculators for TUH, UCLA, and JHU, respectively. Within the validation site of TUH, PCRC-MRI had the highest ROC AUC value for previously published risk calculators at 0.857 while ClarityDX Prostate + MRI + DRE had the highest overall ROC AUC at 0.874. ClarityDX Prostate models with MRI had ROC AUC values ≥ 0.893 for CU which was not significantly different from SPCC which had the highest ROC AUC value of 0.899 for this site.

### Performance of ClarityDX Prostate models for predicting grade group ≥ 2 PCa in the training and validation cohorts

When aggregating sites into training and validation cohorts, all four ClarityDX Prostate models remained the top-performing models in each category of DRE and MRI availability (Table 4). When using a threshold of 25% for predicting grade group ≥2 PCa, ClarityDX Prostate had sensitivity and specificity values of 94% and 34% in the training cohort, respectively, and 95% and 32% in the validation cohort, respectively. Using the same 25% threshold, ClarityDX Prostate +DRE had 3% higher specificity than ClarityDX Prostate for both the training and validation cohorts.

**Table 4:** Risk calculator performance with or without MRI and DRE data for predicting grade group ≥2 prostate cancer by cohort

	Training cohort						Validation cohort					
	ROC AUC	Cutoff	Sensitivity	Specificity	PPV	NPV	ROC AUC	Cutoff	Sensitivity	Specificity	PPV	NPV
	(CI)		% (CI)	% (CI)	% (CI)	% (CI)	(CI)		% (CI)	% (CI)	% (CI)	% (CI)
**No MRI, No DRE**												
PCPTRC	0.66 (0.64–0.68)	≥5.214	94 (92– 95)	17 (15– 19)	45 (43–47)	79 (73–83)	0.73 (0.70–0.76)	≥6.022	94 (91–95)	26 (23–29)	49 (46–52)	84 (79–88)
PBCG	0.70 (0.67–0.72)	≥13.78	94 (92–95)	20 (17–22)	46 (44–48)	81 (76–85)	0.75 (0.73–0.78)	≥16.24	94 (91–95)	22 (19–25)	48 (45–51)	82 (76–87)
ClarityDX Prostate	**0.80 (0.79–0.82)**	≥25	94 (93–96)	**34 (31–37)**	**51 (48–53)**	**89 (86–92)**	**0.81 (0.78–0.83)**	≥25	95 (93–97)	**32 (29–36)**	**52 (49–55)**	**90 (85–93)**
**No MRI, DRE available**												
PCPTRC	0.68 (0.66–0.70)	≥5.393	94 (92–95)	20 (18–22)	46 (44–48)	81 (76–85)	0.75 (0.72–0.78)	≥5.839	94 (91–95)	25 (22–29)	49 (46–52)	84 (78–88)
PBCG	0.71 (0.68–0.73)	≥12.89	94 (92–95)	18 (16–20)	45 (43–48)	79 (74–84)	0.77 (0.74–0.79)	≥14.95	94 (91–95)	22 (19–25)	48 (45–51)	82 (76–87)
ClarityDX Prostate +DRE	**0.82 (0.80–0.83)**	≥25	94 (92–95)	**37 (34–40)**	**52 (50–54)**	**89 (86–91)**	**0.82 (0.80–0.84)**	≥25	95 (93–97)	**35 (32–39)**	**53 (50–56)**	**91 (87–94)**
**MRI available, No DRE**												
Radtke	0.84 (0.82–0.86)	≥33.24	95 (93–96)	32 (29–35)	50 (47–53)	89 (86–92)	0.84 (0.80–0.88)	≥31.49	95 (90–98)	27 (22–33)	42 (36–47)	91 (82–96)
Mehralivand	0.84 (0.82–0.86)	≥48.93	95 (93–96)	35 (32–38)	51 (48–54)	90 (87–93)	0.84 (0.79–0.88)	≥43.29	95 (90–98)	33 (27–39)	44 (38–49)	92 (84–96)
PLUM	0.84 (0.82–0.86)	≥5.899	95 (93–96)	37 (34–40)	52 (49–54)	90 (87–93)	0.85 (0.80–0.89)	≥3.692	95 (90–98)	30 (24–35)	42 (37–48)	91 (83–96)
Imperial RAPID	0.84 (0.82–0.86)	≥10.77	95 (93–96)	31 (28–34)	49 (47–52)	89 (85–92)	0.85 (0.80–0.89)	≥10.84	95 (90–98)	39 (33–45)	46 (40–52)	93 (86–97)
Leeuwen	0.83 (0.80–0.85)	≥0.7542	95 (93–96)	25 (22–28)	47 (45–50)	86 (82–90)	0.85 (0.80–0.89)	≥4.958	95 (90–98)	30 (24–36)	43 (37–48)	91 (83–96)
PCRC-MRI	0.85 (0.83–0.87)	≥13.62	95 (93–96)	39 (36–42)	53 (50–56)	91 (88–93)	0.86 (0.81–0.89)	≥11.45	95 (90–98)	40 (34–46)	46 (41–52)	93 (87–97)
ClarityDX Prostate +MRI	**0.89 (0.87–0.91)**	≥17	96 (95–97)	**45 (42–49)**	**56 (53–58)**	**94 (92–96)**	**0.87 (0.83–0.91)**	≥17	95 (90–98)	**44 (38–50)**	**48 (42–54)**	**94 (88–97)**
**MRI available, DRE available**												
Radtke	0.84 (0.82–0.86)	≥35.29	95 (93–96)	34 (31–37)	51 (48–53)	90 (86–92)	0.84 (0.80–0.88)	≥31.49	95 (90–98)	26 (21–32)	41 (36–47)	90 (81–96)
Mehralivand	0.85 (0.83–0.87)	≥49.23	95 (93–96)	34 (32–38)	51 (48–54)	90 (87–93)	0.84 (0.79–0.88)	≥43.29	95 (90–98)	32 (26–38)	43 (38–49)	92 (84–96)
PLUM	0.84 (0.82–0.86)	≥5.899	95 (93–96)	37 (34–40)	52 (49–54)	90 (87–93)	0.85 (0.80–0.89)	≥3.692	95 (90–98)	30 (24–36)	42 (37–48)	91 (83–96)
Imperial RAPID	0.84 (0.82–0.86)	≥10.77	95 (93–96)	31 (28–34)	49 (47–52)	89 (85–92)	0.85 (0.80–0.89)	≥10.84	95 (90–98)	39 (33–45)	46 (40–52)	93 (87–97)
Leeuwen	0.83 (0.81–0.85)	≥1.058	95 (93–96)	28 (25–31)	48 (46–51)	88 (84–91)	0.85 (0.80–0.89)	≥4.958	95 (90–98)	30 (24–36)	42 (37–48)	91 (83–96)
PCRC-MRI	0.85 (0.83–0.87)	≥13.98	95 (93–96)	39 (36–42)	53 (50–55)	91 (88–93)	0.86 (0.81–0.89)	≥11.45	95 (90–98)	38 (32–44)	46 (40–52)	93 (86–97)
ClarityDX Prostate + MRI + DRE	**0.89 (0.88–0.91)**	≥17	96 (94–97)	**46 (43–49)**	**56 (53–59)**	**94 (91–96)**	**0.88 (0.83–0.91)**	≥17	95 (90–98)	**47 (40–53)**	**49 (43–55)**	**94 (89–98)**

Highest values for ROC AUC, specificity, PPV, and NPV are bolded in each DRE/MRI category.

When MRI data are available, a 17% threshold for predicting grade group ≥2 PCa allowed ClarityDX Prostate + MRI to have sensitivity and specificity values of 96% and 45% in the training cohort, respectively, and 95% and 44% in the validation cohort, respectively. ClarityDX Prostate + MRI + DRE had similar sensitivity, specificity, positive predictive value, and negative predictive values to ClarityDX Prostate + MRI for both the training and validation cohorts, suggesting that the addition of DRE in the MRI-based model had minimal impact on model performance.

The sensitivity and specificity of ClarityDX Prostate models depend on the chosen threshold for identifying patients with grade group ≥2 PCa. When using a threshold of 10%, ClarityDX Prostate models without MRI identified ≥99.6% of grade group 2 PCa while potentially avoiding ≥2.5% of all biopsies and ≥4.4% of unnecessary biopsies less than grade group 2 in the validation and training cohorts (Supplementary Table S2 and Supplementary Table S3). A 25% threshold could have reduced ≥32% of unnecessary biopsies in the validation and training cohorts while identifying ≥94% of grade group ≥2 PCa.

For ClarityDX Prostate models using MRI data, a threshold of 10% identified ≥97% of grade group 2 PCa in the validation and training cohorts while potentially avoiding ≥14% of all biopsies and ≥23% of unnecessary biopsies less than grade group 2. A 17% threshold could have reduced ≥44% of unnecessary biopsies in the validation and training cohorts while identifying ≥94.8% of grade group ≥2 PCa.

Decision curve analysis was performed to determine the net benefit of ClarityDX Prostate models compared to common clinical tests and models (Supplementary Fig. S3). At nearly all threshold probabilities, all 4 ClarityDX Prostate models provided a greater net benefit than PSA, free PSA ratio, PCPTRC and PBCG risk calculators. The highest PI-RADS score provided a greater net benefit than PSA, free PSA ratio, PCPTRC and PBCG risk calculators at threshold probabilities above 0.14, although ClarityDX Prostate +MRI and ClarityDX Prostate + MRI + DRE models had a greater net benefit than highest PI-RADS score with all threshold probabilities above 0.01. The estimated results of biopsies avoided per 100 patients from decision curve analysis reflected the same rank order of test/model performance as the net benefit results.

### Calibration

**Fig. 1:**
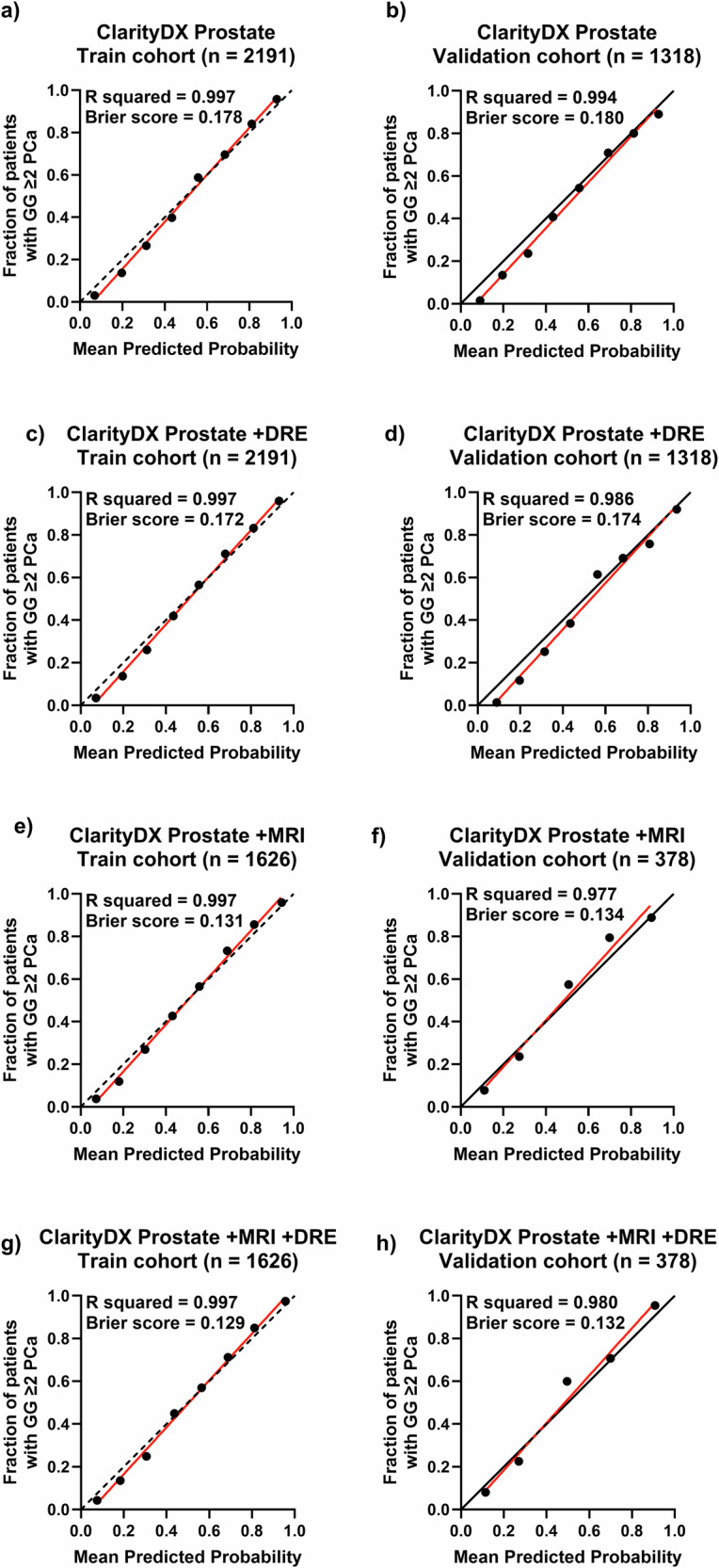
Calibration curves for ClarityDX Prostate models. Training (**a**, **c**, **e**, **g**) and validation (**b**, **d**, **f**, **h**) cohorts when predicting grade group ≥2 prostate cancer. Data points were fitted with linear regression with R squared values and Brier scores shown. Brier scores generally improve as more features are used in models. Graphs created and composited with GraphPad Prism 10.5.0.

All four ClarityDX Prostate models were well calibrated with R squared values of 0.997 in the training cohorts and ≥0.977 in the validation cohorts (Fig. 1). ClarityDX Prostate models without MRI had Brier scores between 0.172 and 0.180 while models using MRI data had lower (better) Brier scores between 0.129 and 0.134. Most calibration curves had a slight sigmoidal shape which is common for random forest models.

### SHAP analysis

**Fig. 2:**
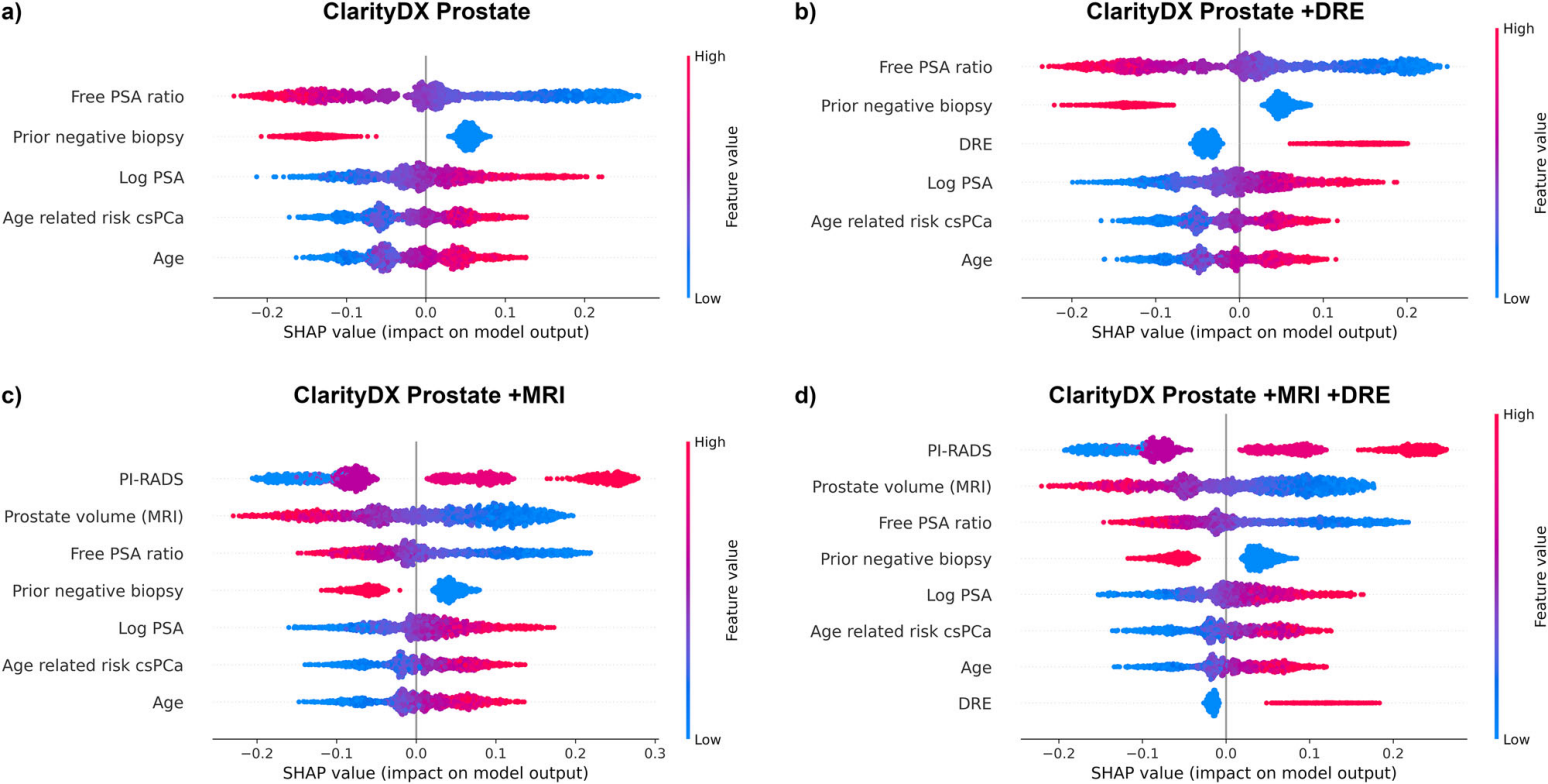
Model interpretability by using SHAP on the training cohorts. SHAP values are shown for ClarityDX Prostate (**a**), ClarityDX Prostate + DRE (**b**), ClarityDX Prostate + MRI (**c**), and ClarityDX Prostate + MRI + DRE (**d**). Each symbol represents the SHAP value for one patient in the training cohort. SHAP values represent how the feature value changed model probability (e.g., +0.1 = +10% model probability for predicting grade group ≥2 prostate cancer). Graphs created with Python’s SHAP library and composited with GIMP 3.0.4.

SHAP analysis was used to interpret ClarityDX Prostate models which are ensembles of calibrated non-linear random forest models. SHAP values for each feature for each patient in the training cohort for all 4 ClarityDX Prostate models are shown in Fig. 2. PSA, age, PI-RADS, and DRE directly affected model probabilities (higher model probabilities with higher feature values) while prostate volume, free PSA ratio, and prior negative biopsy status inversely affected model probabilities (lower model probabilities with higher feature values). PI-RADS 4 and 5 increased model probabilities while PI-RADS 3 and below decreased model probabilities. Features were ranked by the sum of absolute SHAP values for all patients (Supplementary Fig. S4). For the ClarityDX Prostate + MRI + DRE model, PI-RADS had the largest impact on model output probability (mean change of 12% in model probability due to this feature) followed by prostate volume (8%), free PSA ratio (7%), prior negative biopsy status (5%), PSA (5%), age-related features (4% each), and DRE (3%).

## Discussion

Given the rapid clinical adoption of MRI in the pre-biopsy setting, we hypothesize that tests predicting csPCa would benefit from the incorporation of MRI features. Most published reports show improved diagnostic performance in risk models predicting csPCa after MRI features such as prostate volume and PI-RADS score were incorporated^29,30^. Including MRI features within the ClarityDX Prostate models significantly increased the ROC AUC values in the validation cohort (*p* values < 0.005). The current study did not use any methods to modify or correct ClarityDX Prostate model predictions based on differences in csPCa prevalence in each cohort since such methods cannot be used on new patients from clinics with unknown prevalence of csPCa. Differences in csPCa prevalence between clinical sites will affect model sensitivity, specificity, and calibration. The current study used at least 4 different clinical sites for training and validation of ClarityDX Prostate models which suggests the models generalize well to many different clinics. However, these models may perform differently in some clinic sites and may require site-specific model thresholds for 95% sensitivity in those sites.

All four ClarityDX Prostate models had an ROC AUC of at least 0.80 in the training and validation cohorts, demonstrating that csPCa can be accurately predicted without MRI or DRE data. DRE was moderately useful for models without MRI features and had minor value for models with MRI features (Supplementary Table S1).

The ClarityDX Prostate thresholds were set to provide 95% sensitivity for csPCa which means 5% of patients with csPCa may have a delayed diagnosis. These thresholds are well within the NCCN 2025 guidelines suggesting that biomarker testing in prostate cancer early detection should have <10% missed/delayed csPCa diagnosis^31^. Using the validation cohort statistics, the number of tests needed to avoid an unnecessary biopsy (i.e., “Number Needed to Treat”) is 4.8 for ClarityDX Prostate, 4.4 for ClarityDX Prostate Prostate + DRE, 3.5 for ClarityDX Prostate + MRI, and 3.3 for ClarityDX Prostate + MRI + DRE. Since all models have 95% sensitivity, the number of tests needed to cause one csPCa to be missed (i.e., “Number Needed to Harm”) is 56 for all models. Prospective clinical utility studies comparing standard of care with and without ClarityDX Prostate are warranted to determine how ClarityDX Prostate impacts clinical care since physicians/patients do not always follow recommendations and standard of care misses some patients with csPCa.

The specificity of the PSA test, ClarityDX Prostate, ClarityDX Prostate + DRE, ClarityDX Prostate + MRI, and ClarityDX Prostate + MRI + DRE at predicting patients without csPCa is 14%, 32%, 35%, 44%, and 47%, respectively. Therefore, the four ClarityDX Prostate models are approximately 2.3 to 3.4-fold more accurate than PSA for determining patients not requiring prostate biopsies.SHAP analysis of ClarityDX Prostate models showed that the MRI features, including PI-RADS and prostate volume, had the greatest impact on model probabilities. For non-MRI features, free PSA ratio had the greatest impact on all ClarityDX Prostate model probabilities. The greater impact of free PSA ratio over total PSA in ClarityDX Prostate models is interesting given that free PSA is not regularly used in many clinics for PCa screening.

The value of DRE was significantly reduced when MRI data were available since 1) the SHAP value for DRE was half in the ClarityDX Prostate + MRI + DRE model relative to ClarityDX Prostate +DRE model, and 2) the ROC AUC values for ClarityDX Prostate + MRI + DRE and ClarityDX Prostate +MRI (without DRE) were within 0.01 in the validation cohort and the same value in the training cohort. Given the stigma of DRE for patients and physicians, foregoing the DRE and using the ClarityDX Prostate + MRI model may improve the PCa screening experience with minimal harm to patients. In resource-limited clinical settings, the DRE is a low cost and rapid procedure useful for stratifying patients at high-risk for csPCa. As such, the NCCN 2025 guidelines still recommends DRE as part of standard of care for the early detection of prostate cancer^31^.

PCa screening with a PSA test can reduce PCa morbidity and mortality, however, using PSA alone can cause a significant burden of overdiagnosis and overtreatment^2,7^. Use of adjunctive tests such as mpMRI, risk calculators, and biofluid biomarker tests, after an elevated PSA test but prior to a prostate biopsy, may improve the cost-effectiveness of PCa screening by 1) reducing unnecessary prostate biopsies and their associated morbidity, 2) reducing diagnosis and treatment of indolent PCa, and 3) improving the diagnosis of csPCa^6,32–34^. Different “omic”-based prostate cancer tests have been recently developed. Many of these tests, such as Oncotype DX Genomic Prostate Score, Decipher, and Prolaris, assess prostate cancer aggressiveness post-diagnosis^35^ thus they do not have the same intended use as ClarityDX Prostate for predicting grade group ≥2 prostate cancer on biopsy in patients without a diagnosis for prostate cancer. “Omics”-based prostate cancer tests with a similar intended use as ClarityDX Prostate include ExoDx Prostate Test, which analyzes 3 exosomal RNA markers, and STHLM3, which analyzes five proteins and over 100 genetic markers. The ExoDx Prostate Test and STHLM3 can predict grade group ≥2 prostate cancer on biopsy with AUC values ranging from 0.70–0.77 and 0.77–0.82 respectively, which are equal to or below the AUC values of ClarityDX Prostate without MRI^36–40^. Another omics test, Galleri by GRAIL, is an example of a blood-based multicancer early detection tests designed to evaluate people with nonspecific signs and symptoms. Galleri uses machine learning to analyze differences in the cell-free DNA of healthy and cancer cells. Its overall sensitivity for all cancers is 51.5% and specificity is 95.5%. However, for prostate cancer the test correctly identified just 11% of the men with prostate cancer, missing most men with localized disease^41^. Future external validation studies are required to compare these tests in the same cohorts.

The strengths of this study include 1) the use of a contemporary cohort of men recruited over multiple years up to 2024 ranging from six clinical sites spanning three countries, 2) the comparison of 13 different PCa risk calculators for five different clinical sites, and 3) the derivation of new risk models providing higher accuracy for csPCa than all tested publicly available risk calculators.

A limitation of this study includes the low ethnic diversity with 64% to 85% of participants self-identifying as Caucasian in all clinical sites that provided ethnicity data. In the training cohort, 7.1% of participants identified as African American, which is lower than the 12.1% of the U.S. population identifying as African American or Black (https://data.census.gov/table/ACSDT1Y2023.B02001). Additionally, 5.6% of participants identified as Asian, closely reflecting the 6.0% of the U.S. population identifying as Asian. The validation cohort demonstrated substantially less ethnic diversity, as most patients were recruited from Czechia, a population that is predominantly Caucasian. Further studies are warranted to validate ClarityDX Prostate models in more ethnically diverse populations.

In patients suspected of PCa, the decision to perform a prostate biopsy hinges on available clinical data. Although MRI and DRE data can be informative, they may not be available. The ClarityDX Prostate models derived in this study are highly accurate for csPCa and, as recommended by the AUA, EAU, and CUA, can be used as adjunctive PCa screening tools by family physicians or urologists who may or may not have experience with DRE or access to mpMRI.

## Methods

### Study design

Observational data were aggregated from cohorts in six organizations including the University of California (UCLA), Los Angeles, USA, Johns Hopkins University (JHU), Baltimore, USA, University of Calgary (UC), Canada, University of Alberta (UA), Edmonton, Canada, Thomayer University Hospital (TUH), Prague, Czechia, and Chesapeake Urology (CU), Maryland, USA. Eligibility requirements included patients 1) undergoing a prostate biopsy due to an elevated PSA or palpable DRE abnormality, 2) without a prior PCa diagnosis, and 3) having available data for model inputs including age, serum-based total and free PSA, as well as prostate volume from MRI and PI-RADS for models using MRI data. The UA and UC sites had additional eligibility requirements for patients between 40 and 75 years of age with at least 3 ng/mL total PSA results within six months of enrollment and excluded patients with a prior cancer diagnosis that was not non-melanoma skin cancer. Prostate biopsies from all six organizations were performed between September 2009 and August 2024.

Models without MRI were created using data from 2191 eligible patients from UCLA, UC, and JHU (training cohort) out of a total of 2234 patients from these sites. After derivation, models were fixed and subsequently validated on 1318 patients from UA, TUH, and CU (validation cohort) out of a total of 1763 patients from these sites (Supplementary Fig. S1).

When deriving and validating models with MRI data, UC and UA were removed from the training and validation cohorts, respectively, since these sites had less than 50% of patients with MRI data. Additionally, the training and validation cohorts for models using MRI data required patients to have MRI prostate ellipsoid volume (length * width * height * π / 6) and highest PI-RADS score, reducing the training and validation cohorts to 1626 and 378 patients, respectively.

### Ethics

The Health Research Ethics Board of Alberta approved the collection of patient data from UC and TUH (HREBA.CC-18-0241) plus UA (19-0109). The UCLA Institutional Review Board approved the collection of patient data from UCLA (IRB #11-001580 and IRB #19-001136). The collection of data from JHU was approved by research project CR00040216. The Institutional Review Board for CU approved the retrospective analysis of patient data (IRB #20235399). Informed consent was obtained from all participants, and the study adhered to the standards set by the Declaration of Helsinki^42^. This observational study did not impact patient management since ClarityDX Prostate test results were not provided to the clinical sites.

### PSA and free PSA tests

Total PSA and free PSA results for patients from UC and UA were frozen, stored, and acquired in batches within 12 weeks using a Roche Cobas e801 system as previously described^28,43^. For UCLA, JHU, TUH, and CU, total PSA and free PSA results were acquired as part of standard clinical practice.

### Prostate biopsies

Prostate biopsies were performed according to the standard procedures for each clinical site. UC and UA primarily used the transrectal ultrasound-guided systematic 12-core biopsies without MRI guidance. TUH took at least 12 systematic cores with a median of 4 additional targeted cores for MRI-identified lesions. Prostate biopsies from UCLA also used 12 systematic cores with 3 to 5 additional targeted cores per MRI-identified lesion using an Artemis system (Eigen). CU performed 12 core systematic biopsies with 2 extra cores per MRI-identified lesion. Pathological assessment of biopsy specimens followed the standard procedures at each clinical site.

### Predictive models and risk calculators

ClarityDX Prostate models were created according to a previous study without any further hyperparameter tuning^28^. Briefly, ensembles of random forest models calibrated with isotonic regression were created for each ClarityDX Prostate model which may or may not have included 1) MRI prostate volume (in cubic centimeters) and highest PI-RADS score ( ± MRI) and 2) digital rectal exam findings ( ± DRE) which could be normal or abnormal due to asymmetry, nodules, or induration (Supplementary Fig. S2). All ClarityDX Prostate models used log PSA, free PSA ratio (free PSA / total PSA), age (years), age-related risk of csPCa, and prior negative biopsy status (yes or no)^28^. All models were trained on the training cohort to predict grade group ≥2 csPCa. Derived models were fixed and evaluated on patients from the validation cohort. Predictive models were created using Python 3.11.5 with scikit-learn (version 1.3.2).

Feature importance for ClarityDX Prostate models was performed using Python’s SHapley Additive exPlanations (SHAP) package (version 0.43.0) which describes how the final model probability was modified by each feature for each patient. Mean absolute SHAP values for all model features within the training cohort provided a ranking for the importance of model features.

ClarityDX Prostate models without MRI were compared to ERSPC-3/4, PCPTRC, and PBCG. Risk calculator predictions were obtained from the ERSPC-3/4 web application (https://www.prostatecancer-riskcalculator.com/seven-prostate-cancer-risk-calculators) and PCPTRC and PBCG source code (https://github.com/ClevelandClinicQHS/riskcalc-website/tree/main/PCPTRC, https://github.com/ClevelandClinicQHS/riskcalc-website/tree/main/PBCG).

ClarityDX Prostate models using MRI features were compared with up to 10 other MRI-based PCa risk calculators as described in Supplementary Table S1. The following models were recreated in Python using the intercepts and coefficients from their publications: Mehralivand, Radtke, PLUM, Imperial RAPID, Leeuwen, and PCRC-MRI^8,19,21–26,44–46^. Predictions from the following models were obtained using online web applications: MSP-RC (https://darasriskcalcs.shinyapps.io/MSP-RC/), ERSPC-3/4 MRI (https://www.prostatecancer-riskcalculator.com/seven-prostate-cancer-risk-calculators), SPCC (https://med.stanford.edu/ucil/nomogram.html), BCN2RC (https://mripcaprediction.shinyapps.io/MRIPCaPrediction/).

When DRE was unavailable, DRE was input as normal for risk calculators and ClarityDX Prostate models using DRE. Web-based risk calculators were not applied to patient data from UCLA due to this data being in a dedicated environment at UCLA Health called ULEAD which did not have internet access.

### Statistical analyses

Model statistics and model calibration curve values were calculated using Python 3.11.5 using scikit-learn (1.3.2), scipy (1.11.3), and scikits.bootstrap (1.1.0) packages. ROC AUC values were calculated using scikit-learn’s roc_auc_score function. When comparing patients with and without csPCa, continuous and categorical features were compared with Mann-Whitney U-tests and Fisher’s exact tests, respectively, with statistical significance determined by *p* values ≤ 0.05. DeLong’s method was used to compare ROC curves^47^. All *p* values were adjusted for multiple comparisons using Holm’s method, as implemented in the Python library statsmodels (0.14.5). Decision curve analysis was performed using the Python library dcurves (1.1.5).

When predicting csPCa with ClarityDX Prostate models, threshold values of 25% and 17% were chosen for models without and with MRI features, respectively, since these thresholds provided approximately 95% sensitivity, which is similar to the sensitivity of PI-RADS ≥ 3 for csPCa^28^. Threshold values for other risk calculators were set independently for each cohort to obtain 95% sensitivity and maximum specificity. Confidence intervals for ROC AUC, sensitivity, specificity, positive predictive value (PPV), and negative predictive value (NPV) were determined with 10,000 resamples of bias-corrected and accelerated bootstrapping.

## Supplementary information

41746_2026_2642_MOESM1_ESM

## Data Availability

The data generated in this study are available within the article and its supplementary material.
