# Supplementary material for: Predicting clinically significant prostate cancer with or without digital rectal exam and MRI data using ClarityDX Prostate models
Source: NPJ Digit Med. 2026 Apr 17;9:467. doi: 10.1038/s41746-026-02642-1 (PMC13273144; doi:10.1038/s41746-026-02642-1)
Supplement: Supplementary file 1 — 41746_2026_2642_MOESM1_ESM [file 41746_2026_2642_MOESM1_ESM.pdf]

Supplementary Material

Predicting clinically significant prostate cancer with or without digital rectal exam and MRI data using ClarityDX Prostate models

Table of Contents

Page

Supplementary Figure S1 ……………………………………………………………………1

Supplementary Figure S2 ……………………………………………………………………2

Supplementary Figure S3 ……………………………………………………………………3

Supplementary Figure S4 ……………………………………………………………………4

Supplementary Table S1 ……………………………………………………………………5

Supplementary Table S2 ……………………………………………………………………6

Supplementary Table S3 ……………………………………………………………………7

Supplementary References ……………………………………………………………………8

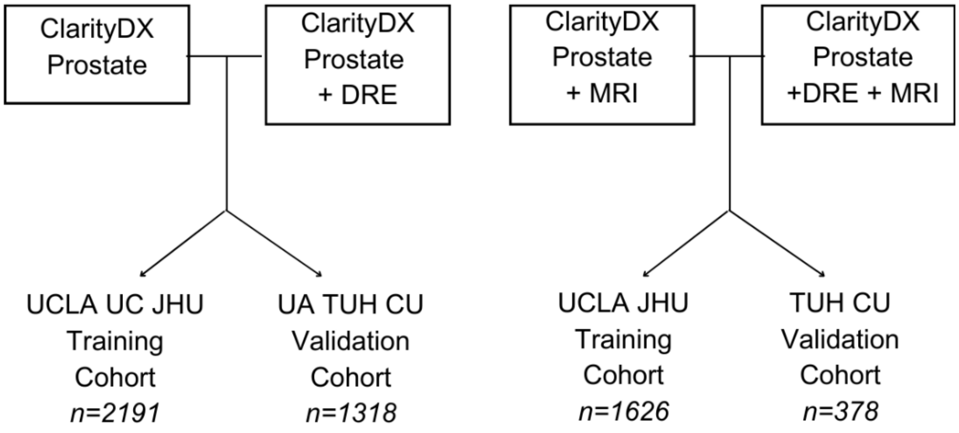

Supplementary Figure S1. Training and validation cohort sizes with and without mpMRI data. Abbreviations include: UCLA: University of California, Los Angeles, JHU: Johns Hopkins University, UC: University of Calgary, UA: University of Alberta, TUH: Thomayer University Hospital, CU: Chesapeake Urology. Figure created with Microsoft PowerPoint 2016.

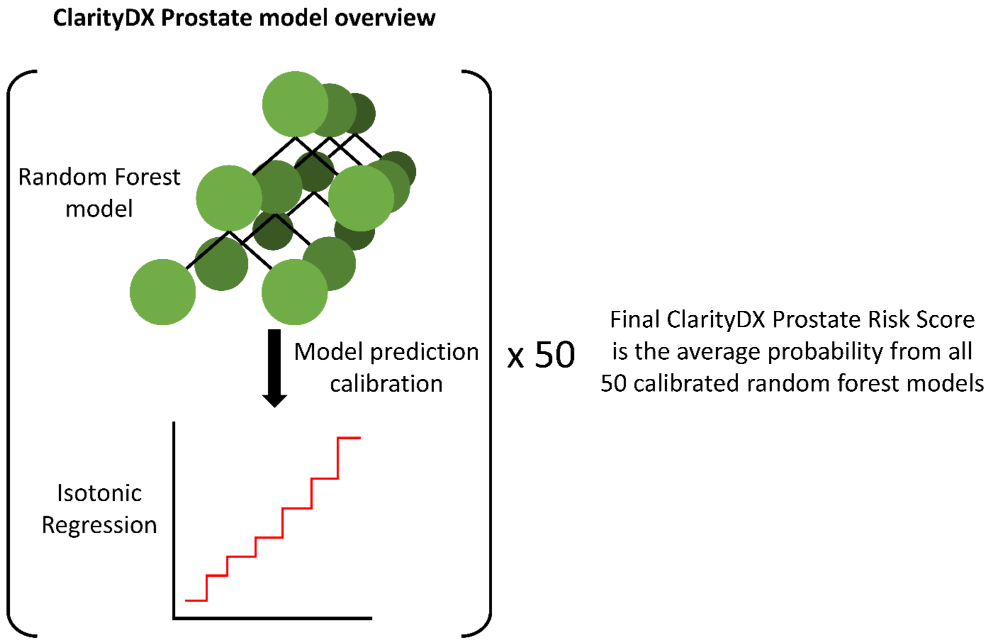

Supplementary Figure S2. ClarityDX Prostate model overview. Figure created with Microsoft PowerPoint 2016.

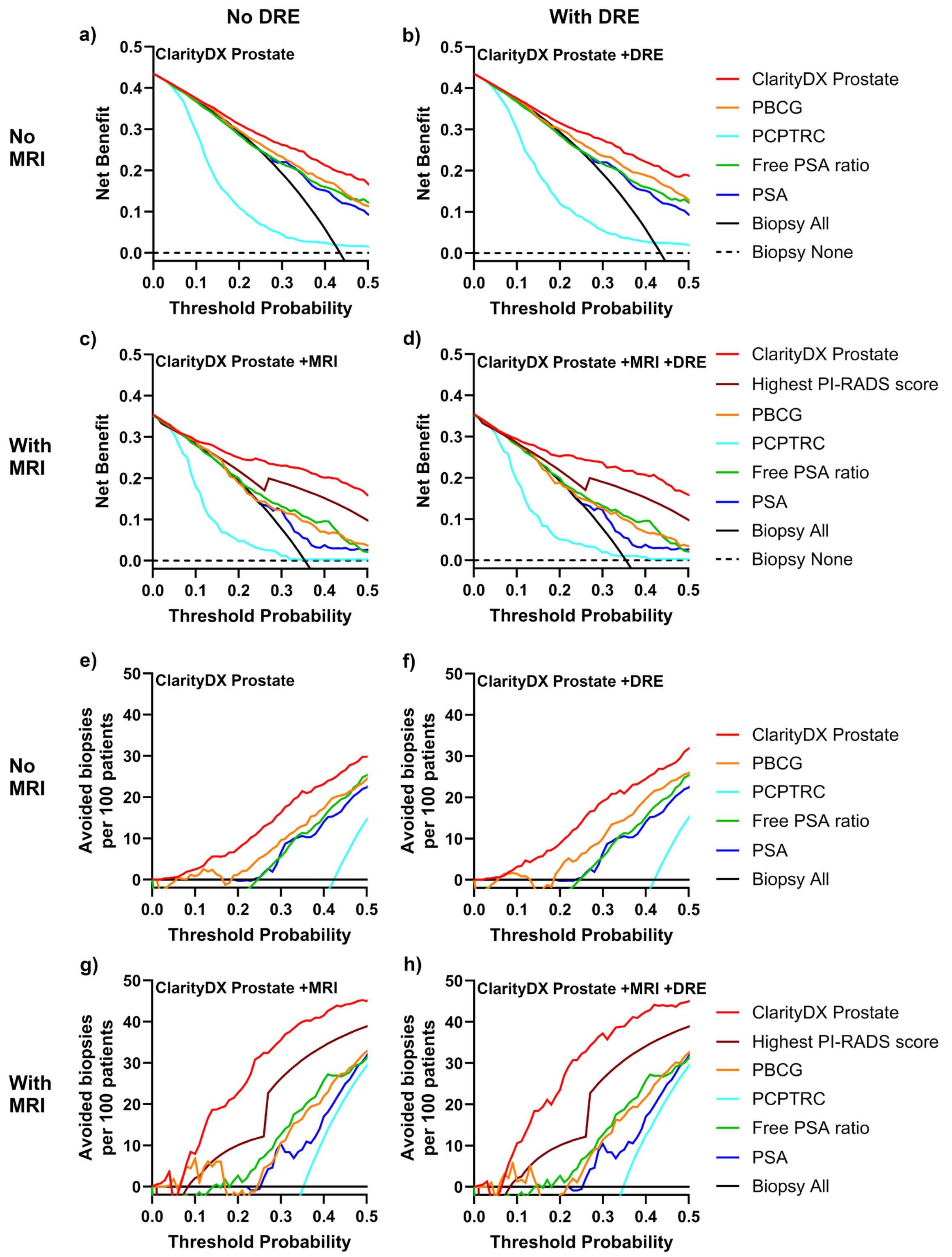

Supplementary Figure S3. Decision Curve Analysis of ClarityDX Prostate models with common clinical tests and risk calculators using validation cohort data. Models include ClarityDX Prostate (a, e), ClarityDX Prostate +DRE (b, f), ClarityDX Prostate +MRI (c, g), and ClarityDX Prostate +MRI +DRE (d, h). Decision Curve Analysis included net benefit (a-d) and avoided biopsies per 100 patients (e-h). Graphs created with GraphPad Prism 10.5.0 and composited with GIMP 3.0.4.

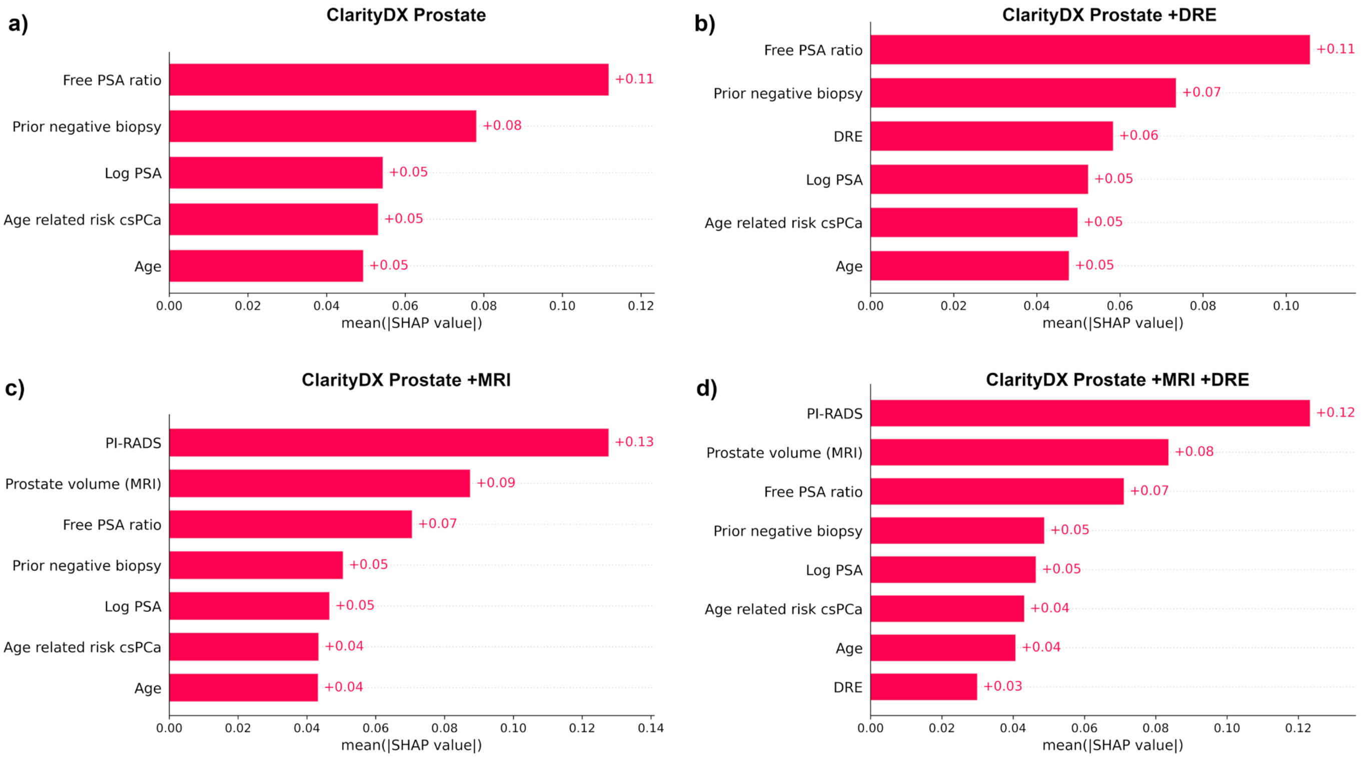

Supplementary Figure S4. Mean absolute SHAP values for ClarityDX Prostate models which did not include DRE (a, c), included DRE (b, d), did not include MRI data (a, b), or included MRI data (c, d). SHAP values were determined with the training cohorts. Features with higher mean absolute SHAP values had a greater impact on changing model probabilities. Graphs created with Python's shap library and composited with GIMP 3.0.4.

Supplementary Table S1. Characteristics of models predicting grade group ≥2 prostate cancer.

	Features used by models									Clinical sites		
**Risk calculator**	**PSA**	**Free PSA**	**Age**	**Ethnicity**	**Family History PCa**	**PNB**	**DRE**	**PI-RADS score**	**Prostate volume**	**ClarityDX Training**	**ClarityDX Validation**	**Reference**
ERSPC-3/4	✓	×	×	×	×	✓	✓	×	×	-	-	[1]
PCPTRC	✓	×	✓	✓	✓	✓	✓	×	×	-	-	[2]
PBCG	✓	×	✓	✓	✓	✓	✓	×	×	-	-	[3]
MSP-RC	✓	×	✓	✓	✓	✓	✓	✓	✓	-	-	[4]
ERSPC-3/4 MRI	✓	×	✓	×	×	✓	✓	✓	✓	-	-	[5]
SPCC	✓	×	✓	✓	×	✓	×	✓	✓	-	-	[6]
Mehralivand	✓	×	✓	✓	×	✓	✓	✓	✓	-	-	[7]
Radtke	✓	×	✓	×	×	✓	✓	✓	✓	-	-	[8]
PLUM	✓	×	✓	✓	✓	✓	×	✓	✓	-	-	[9]
Imperial RAPID	✓	×	✓	×	×	✓	×	✓	✓	-	-	[10]
BCN2RC	✓	×	✓	×	✓	✓	✓	✓	✓	-	-	[11]
Leeuwen	✓	×	✓	×	×	✓	✓	✓	✓	-	-	[12]
PCRC-MRI	✓	×	✓	✓	×	✓	✓	✓	✓	-	-	[13]
ClarityDX Prostate	✓	✓	✓	×	×	✓	×	×	×	UCLA, JHU, UC	UA, TUH, CU	Current study
ClarityDX Prostate +DRE	✓	✓	✓	×	×	✓	✓	×	×	UCLA, JHU, UC	UA, TUH, CU	[14]
ClarityDX Prostate +MRI	✓	✓	✓	×	×	✓	×	✓	✓	UCLA, JHU	TUH, CU	Current study
ClarityDX Prostate +MRI +DRE	✓	✓	✓	×	×	✓	✓	✓	✓	UCLA, JHU	TUH, CU	Current study

PSA: Prostate specific antigen
PNB: Prior negative biopsy status
DRE: Digital rectal exam findings
PI-RADS: Prostate Imaging Reporting and Data System
UCLA: University of California, Los Angeles
JHU: Johns Hopkins University
UC: University of Calgary
UA: University of Alberta
TUH: Thomayer University Hospital
CU: Chesapeake Urology

Supplementary Table S2. Prostate cancers found, missed, and biopsies avoided using ClarityDX Prostate models in the training cohort.

Thresholds	GG ≥1 PCa found n (%)	GG ≥1 PCa missed n (%)	GG ≥2 PCa found n (%)	GG ≥2 PCa missed n (%)	GG ≥3 PCa found n (%)	GG ≥3 PCa missed n (%)	GG ≥4 PCa found n (%)	GG ≥4 PCa missed n (%)	GG 5 PCa found n (%)	GG 5 PCa missed n (%)	Biopsies avoided* n (%)	Unnecessary biopsies avoided** n (%)
**ClarityDX Prostate**												
5	1384 (99.4%)	9 (0.6%)	920 (99.9%)	1 (0.1%)	451 (99.8%)	1 (0.2%)	267 (99.6%)	1 (0.4%)	162 (100.0%)	0 (0.0%)	43 (2.0%)	42 (3.3%)
10	1370 (98.3%)	23 (1.7%)	917 (99.6%)	4 (0.4%)	451 (99.8%)	1 (0.2%)	267 (99.6%)	1 (0.4%)	162 (100.0%)	0 (0.0%)	98 (4.5%)	94 (7.4%)
15	1348 (96.8%)	45 (3.2%)	913 (99.1%)	8 (0.9%)	450 (99.6%)	2 (0.4%)	266 (99.3%)	2 (0.7%)	161 (99.4%)	1 (0.6%)	169 (7.7%)	161 (12.7%)
20	1285 (92.2%)	108 (7.8%)	891 (96.7%)	30 (3.3%)	445 (98.5%)	7 (1.5%)	263 (98.1%)	5 (1.9%)	160 (98.8%)	2 (1.2%)	315 (14.4%)	285 (22.4%)
25	1217 (87.4%)	176 (12.6%)	869 (94.4%)	52 (5.6%)	438 (96.9%)	14 (3.1%)	259 (96.6%)	9 (3.4%)	158 (97.5%)	4 (2.5%)	483 (22.0%)	431 (33.9%)
30	1134 (81.4%)	259 (18.6%)	836 (90.8%)	85 (9.2%)	426 (94.2%)	26 (5.8%)	252 (94.0%)	16 (6.0%)	154 (95.1%)	8 (4.9%)	666 (30.4%)	581 (45.7%)
**ClarityDX Prostate +DRE**												
5	1381 (99.1%)	12 (0.9%)	919 (99.8%)	2 (0.2%)	451 (99.8%)	1 (0.2%)	267 (99.6%)	1 (0.4%)	162 (100%)	0 (0%)	43 (2.0%)	41 (3.2%)
10	1368 (98.2%)	25 (1.8%)	918 (99.7%)	3 (0.3%)	451 (99.8%)	1 (0.2%)	267 (99.6%)	1 (0.4%)	162 (100%)	0 (0%)	104 (4.7%)	101 (8.0%)
15	1339 (96.1%)	54 (3.9%)	911 (98.9%)	10 (1.1%)	450 (99.6%)	2 (0.4%)	266 (99.3%)	2 (0.7%)	162 (100%)	0 (0%)	183 (8.4%)	173 (13.6%)
20	1278 (91.7%)	115 (8.3%)	891 (96.7%)	30 (3.3%)	447 (98.9%)	5 (1.1%)	264 (98.5%)	4 (1.5%)	161 (99.4%)	1 (0.6%)	332 (15.2%)	302 (23.8%)
25	1195 (85.8%)	198 (14.2%)	863 (93.7%)	58 (6.3%)	436 (96.5%)	16 (3.5%)	259 (96.6%)	9 (3.4%)	158 (97.5%)	4 (2.5%)	529 (24.1%)	471 (37.1%)
30	1095 (78.6%)	298 (21.4%)	821 (89.1%)	100 (10.9%)	424 (93.8%)	28 (6.2%)	252 (94.0%)	16 (6.0%)	154 (95.1%)	8 (4.9%)	742 (33.9%)	642 (50.6%)
**ClarityDX Prostate +MRI**												
5	977 (98.6%)	14 (1.4%)	678 (100.0%)	0 (0.0%)	368 (100.0%)	0 (0.0%)	229 (100.0%)	0 (0.0%)	136 (100.0%)	0 (0.0%)	85 (5.2%)	85 (9.0%)
10	934 (94.2%)	57 (5.8%)	671 (99.0%)	7 (1.0%)	365 (99.2%)	3 (0.8%)	227 (99.1%)	2 (0.9%)	136 (100.0%)	0 (0.0%)	223 (13.7%)	216 (22.8%)
15	883 (89.1%)	108 (10.9%)	660 (97.3%)	18 (2.7%)	363 (98.6%)	5 (1.4%)	226 (98.7%)	3 (1.3%)	136 (100.0%)	0 (0.0%)	391 (24.0%)	373 (39.3%)
17	857 (86.5%)	134 (13.5%)	652 (96.2%)	26 (3.8%)	361 (98.1%)	7 (1.9%)	225 (98.3%)	4 (1.7%)	135 (99.3%)	1 (0.7%)	456 (28.0%)	430 (45.4%)
20	815 (82.2%)	176 (17.8%)	638 (94.1%)	40 (5.9%)	359 (97.6%)	9 (2.4%)	224 (97.8%)	5 (2.2%)	134 (98.5%)	2 (1.5%)	545 (33.5%)	505 (53.3%)
25	782 (78.9%)	209 (21.1%)	627 (92.5%)	51 (7.5%)	354 (96.2%)	14 (3.8%)	222 (96.9%)	7 (3.1%)	134 (98.5%)	2 (1.5%)	640 (39.4%)	589 (62.1%)
30	738 (74.5%)	253 (25.5%)	606 (89.4%)	72 (10.6%)	350 (95.1%)	18 (4.9%)	221 (96.5%)	8 (3.5%)	133 (97.8%)	3 (2.2%)	740 (45.5%)	668 (70.5%)
**ClarityDX Prostate +MRI +DRE**												
5	976 (98.5%)	15 (1.5%)	678 (100.0%)	0 (0.0%)	368 (100.0%)	0 (0.0%)	229 (100.0%)	0 (0.0%)	136 (100.0%)	0 (0.0%)	85 (5.2%)	85 (9.0%)
10	927 (93.5%)	64 (6.5%)	668 (98.5%)	10 (1.5%)	365 (99.2%)	3 (0.8%)	227 (99.1%)	2 (0.9%)	136 (100.0%)	0 (0.0%)	236 (14.5%)	226 (23.8%)
15	874 (88.2%)	117 (11.8%)	660 (97.3%)	18 (2.7%)	363 (98.6%)	5 (1.4%)	226 (98.7%)	3 (1.3%)	136 (100.0%)	0 (0.0%)	398 (24.5%)	380 (40.1%)
17	849 (85.7%)	142 (14.3%)	649 (95.7%)	29 (4.3%)	362 (98.4%)	6 (1.6%)	226 (98.7%)	3 (1.3%)	136 (100.0%)	0 (0.0%)	462 (28.4%)	433 (45.7%)
20	814 (82.1%)	177 (17.9%)	640 (94.4%)	38 (5.6%)	359 (97.6%)	9 (2.4%)	224 (97.8%)	5 (2.2%)	135 (99.3%)	1 (0.7%)	545 (33.5%)	507 (53.5%)
25	774 (78.1%)	217 (21.9%)	620 (91.4%)	58 (8.6%)	353 (95.9%)	15 (4.1%)	222 (96.9%)	7 (3.1%)	134 (98.5%)	2 (1.5%)	653 (40.2%)	595 (62.8%)
30	740 (74.7%)	251 (25.3%)	605 (89.2%)	73 (10.8%)	349 (94.8%)	19 (5.2%)	220 (96.1%)	9 (3.9%)	133 (97.8%)	3 (2.2%)	742 (45.6%)	669 (70.6%)

GG: Grade group
PCa: Prostate cancer
DRE: Digital rectal exam findings
MRI: Availability of PI-RADS score and prostate volume from magnetic resonance imaging
* Patients receiving a biopsy but had a ClarityDX Prostate Risk Score below the threshold
** Patients with a negative biopsy or grade group 1 PCa with a ClarityDX Prostate Risk Score below the threshold.

The number of PCa found is the number of true positive results
The number of PCa missed is the number of false negative results
The percentage of PCa found is the sensitivity (%) of ClarityDX Prostate models
The percentage of PCa missed is 100% - sensitivity (%) of ClarityDX Prostate models

Supplementary Table S3. Prostate cancers found, missed, and biopsies avoided using ClarityDX Prostate models in the validation cohort.

Thresholds	GG ≥1 PCa found n (%)	GG ≥1 PCa missed n (%)	GG ≥2 PCa found n (%)	GG ≥2 PCa missed n (%)	GG ≥3 PCa found n (%)	GG ≥3 PCa missed n (%)	GG ≥4 PCa found n (%)	GG ≥4 PCa missed n (%)	GG 5 PCa found n (%)	GG 5 PCa missed n (%)	Biopsies avoided* n (%)	Unnecessary biopsies avoided** n (%)
**ClarityDX Prostate**												
5	853 (99.9%)	1 (0.1%)	573 (100.0%)	0 (0.0%)	225 (100.0%)	0 (0.0%)	88 (100.0%)	0 (0.0%)	50 (100.0%)	0 (0.0%)	7 (0.5%)	7 (0.9%)
10	844 (98.8%)	10 (1.2%)	573 (100.0%)	0 (0.0%)	225 (100.0%)	0 (0.0%)	88 (100.0%)	0 (0.0%)	50 (100.0%)	0 (0.0%)	33 (2.5%)	33 (4.4%)
15	827 (96.8%)	27 (3.2%)	569 (99.3%)	4 (0.7%)	224 (99.6%)	1 (0.4%)	87 (98.9%)	1 (1.1%)	49 (98.0%)	1 (2.0%)	101 (7.7%)	97 (13.0%)
20	802 (93.9%)	52 (6.1%)	560 (97.7%)	13 (2.3%)	219 (97.3%)	6 (2.7%)	85 (96.6%)	3 (3.4%)	48 (96.0%)	2 (4.0%)	164 (12.4%)	151 (20.3%)
25	763 (89.3%)	91 (10.7%)	545 (95.1%)	28 (4.9%)	215 (95.6%)	10 (4.4%)	83 (94.3%)	5 (5.7%)	48 (96.0%)	2 (4.0%)	267 (20.3%)	239 (32.1%)
30	715 (83.7%)	139 (16.3%)	528 (92.1%)	45 (7.9%)	208 (92.4%)	17 (7.6%)	81 (92.0%)	7 (8.0%)	46 (92.0%)	4 (8.0%)	363 (27.5%)	318 (42.7%)
**ClarityDX Prostate +DRE**												
5	853 (99.9%)	1 (0.1%)	573 (100.0%)	0 (0.0%)	225 (100.0%)	0 (0.0%)	88 (100.0%)	0 (0.0%)	50 (100.0%)	0 (0.0%)	8 (0.6%)	8 (1.1%)
10	843 (98.7%)	11 (1.3%)	573 (100.0%)	0 (0.0%)	225 (100.0%)	0 (0.0%)	88 (100.0%)	0 (0.0%)	50 (100.0%)	0 (0.0%)	42 (3.2%)	42 (5.6%)
15	825 (96.6%)	29 (3.4%)	568 (99.1%)	5 (0.9%)	223 (99.1%)	2 (0.9%)	87 (98.9%)	1 (1.1%)	50 (100.0%)	0 (0.0%)	106 (8.0%)	101 (13.6%)
20	808 (94.6%)	46 (5.4%)	562 (98.1%)	11 (1.9%)	220 (97.8%)	5 (2.2%)	86 (97.7%)	2 (2.3%)	49 (98.0%)	1 (2.0%)	171 (13.0%)	160 (21.5%)
25	758 (88.8%)	96 (11.2%)	547 (95.5%)	26 (4.5%)	216 (96.0%)	9 (4.0%)	85 (96.6%)	3 (3.4%)	49 (98.0%)	1 (2.0%)	288 (21.9%)	262 (35.2%)
30	713 (83.5%)	141 (16.5%)	533 (93.0%)	40 (7.0%)	212 (94.2%)	13 (5.8%)	84 (95.5%)	4 (4.5%)	48 (96.0%)	2 (4.0%)	385 (29.2%)	345 (46.3%)
**ClarityDX Prostate +MRI**												
5	185 (97.4%)	5 (2.6%)	132 (98.5%)	2 (1.5%)	77 (100.0%)	0 (0.0%)	47 (100.0%)	0 (0.0%)	19 (100.0%)	0 (0.0%)	23 (6.1%)	21 (8.6%)
10	178 (93.7%)	12 (6.3%)	131 (97.8%)	3 (2.2%)	77 (100.0%)	0 (0.0%)	47 (100.0%)	0 (0.0%)	19 (100.0%)	0 (0.0%)	60 (15.9%)	57 (23.4%)
15	171 (90.0%)	19 (10.0%)	129 (96.3%)	5 (3.7%)	76 (98.7%)	1 (1.3%)	47 (100.0%)	0 (0.0%)	19 (100.0%)	0 (0.0%)	104 (27.5%)	99 (40.6%)
17	167 (87.9%)	23 (12.1%)	127 (94.8%)	7 (5.2%)	75 (97.4%)	2 (2.6%)	46 (97.9%)	1 (2.1%)	19 (100.0%)	0 (0.0%)	115 (30.4%)	108 (44.3%)
20	162 (85.3%)	28 (14.7%)	123 (91.8%)	11 (8.2%)	74 (96.1%)	3 (3.9%)	45 (95.7%)	2 (4.3%)	19 (100.0%)	0 (0.0%)	141 (37.3%)	130 (53.3%)
25	147 (77.4%)	43 (22.6%)	118 (88.1%)	16 (11.9%)	72 (93.5%)	5 (6.5%)	44 (93.6%)	3 (6.4%)	18 (94.7%)	1 (5.3%)	183 (48.4%)	167 (68.4%)
30	133 (70.0%)	57 (30.0%)	111 (82.8%)	23 (17.2%)	68 (88.3%)	9 (11.7%)	43 (91.5%)	4 (8.5%)	17 (89.5%)	2 (10.5%)	211 (55.8%)	188 (77.0%)
**ClarityDX Prostate +MRI +DRE**												
5	184 (96.8%)	6 (3.2%)	132 (98.5%)	2 (1.5%)	77 (100.0%)	0 (0.0%)	47 (100.0%)	0 (0.0%)	19 (100.0%)	0 (0.0%)	23 (6.1%)	21 (8.6%)
10	180 (94.7%)	10 (5.3%)	132 (98.5%)	2 (1.5%)	77 (100.0%)	0 (0.0%)	47 (100.0%)	0 (0.0%)	19 (100.0%)	0 (0.0%)	60 (15.9%)	58 (23.8%)
15	172 (90.5%)	18 (9.5%)	129 (96.3%)	5 (3.7%)	76 (98.7%)	1 (1.3%)	47 (100.0%)	0 (0.0%)	19 (100.0%)	0 (0.0%)	103 (27.2%)	98 (40.2%)
17	167 (87.9%)	23 (12.1%)	127 (94.8%)	7 (5.2%)	75 (97.4%)	2 (2.6%)	46 (97.9%)	1 (2.1%)	19 (100.0%)	0 (0.0%)	121 (32.0%)	114 (46.7%)
20	159 (83.7%)	31 (16.3%)	122 (91.0%)	12 (9.0%)	74 (96.1%)	3 (3.9%)	45 (95.7%)	2 (4.3%)	19 (100.0%)	0 (0.0%)	149 (39.4%)	137 (56.1%)
25	141 (74.2%)	49 (25.8%)	116 (86.6%)	18 (13.4%)	70 (90.9%)	7 (9.1%)	44 (93.6%)	3 (6.4%)	18 (94.7%)	1 (5.3%)	194 (51.3%)	176 (72.1%)
30	134 (70.5%)	56 (29.5%)	112 (83.6%)	22 (16.4%)	68 (88.3%)	9 (11.7%)	43 (91.5%)	4 (8.5%)	17 (89.5%)	2 (10.5%)	214 (56.6%)	192 (78.7%)

GG: Grade group
PCa: Prostate cancer
DRE: Digital rectal exam findings
MRI: Availability of PI-RADS score and prostate volume from magnetic resonance imaging
* Patients receiving a biopsy but had a ClarityDX Prostate Risk Score below the threshold
** Patients with a negative biopsy or grade group 1 PCa with a ClarityDX Prostate Risk Score below the threshold

The number of PCa found is the number of true positive results
The number of PCa missed is the number of false negative results
The percentage of PCa found is the sensitivity (%) of ClarityDX Prostate models
The percentage of PCa missed is 100% - sensitivity (%) of ClarityDX Prostate models

References

1 Roobol, M. J. *et al.* Prediction of Prostate Cancer Risk: The Role of Prostate Volume and Digital Rectal Examination in the ERSPC Risk Calculators. *European Urology* **61**, 577-583 (2012). https://doi.org/10.1016/j.eururo.2011.11.012

2 Ankerst, D. P. *et al.* Prostate cancer prevention trial risk calculator 2.0 for the prediction of low- versus high-grade prostate cancer. *Urology* **83**, 1362-1368 (2014). https://doi.org/10.1016/j.urology.2014.02.035

3 Ankerst, D. P. *et al.* A Contemporary Prostate Biopsy Risk Calculator Based on Multiple Heterogeneous Cohorts. *European urology* **74**, 197-203 (2018). https://doi.org/10.1016/j.eururo.2018.05.003

4 Parekh, S. *et al.* The Mount Sinai Prebiopsy Risk Calculator for Predicting any Prostate Cancer and Clinically Significant Prostate Cancer: Development of a Risk Predictive Tool and Validation with Advanced Neural Networking, Prostate Magnetic Resonance Imaging Outcome Database, and European Randomized Study of Screening for Prostate Cancer Risk Calculator. *European urology open science* **41**, 45-54 (2022). https://doi.org/10.1016/j.euros.2022.04.017

5 Alberts, A. R. *et al.* Prediction of High-grade Prostate Cancer Following Multiparametric Magnetic Resonance Imaging: Improving the Rotterdam European Randomized Study of Screening for Prostate Cancer Risk Calculators. *European Urology* **75**, 310-318 (2019). https://doi.org/10.1016/j.eururo.2018.07.031

6 Wang, N. N. *et al.* The stanford prostate cancer calculator: Development and external validation of online nomograms incorporating PIRADS scores to predict clinically significant prostate cancer. *Urol Oncol* **39**, 831.e819-831.e827 (2021). https://doi.org/10.1016/j.urolonc.2021.06.004

7 Mehralivand, S. *et al.* A Magnetic Resonance Imaging-Based Prediction Model for Prostate Biopsy Risk Stratification. *JAMA oncology* **4**, 678-685 (2018). https://doi.org/10.1001/jamaoncol.2017.5667

8 Radtke, J. P. *et al.* Combined Clinical Parameters and Multiparametric Magnetic Resonance Imaging for Advanced Risk Modeling of Prostate Cancer—Patient-tailored Risk Stratification Can Reduce Unnecessary Biopsies. *European Urology* **72**, 888-896 (2017). https://doi.org/10.1016/j.eururo.2017.03.039

9 Patel, H. D. *et al.* A prostate biopsy risk calculator based on MRI: development and comparison of the Prospective Loyola University multiparametric MRI (PLUM) and Prostate Biopsy Collaborative Group (PBCG) risk calculators. *BJU international* **131**, 227-235 (2023). https://doi.org/10.1111/bju.15835

10 Peters, M. *et al.* Predicting the Need for Biopsy to Detect Clinically Significant Prostate Cancer in Patients with a Magnetic Resonance Imaging–detected Prostate Imaging Reporting and Data System/Likert ≥3 Lesion: Development and Multinational External Validation of the Imperial Rapid Access to Prostate Imaging and Diagnosis Risk Score. *European Urology* **82**, 559-568 (2022). https://doi.org/10.1016/j.eururo.2022.07.022

11 Morote, J. *et al.* The Barcelona Predictive Model of Clinically Significant Prostate Cancer. *Cancers* **14**, 1589 (2022). https://doi.org/10.3390/cancers14061589

12 van Leeuwen, P. J. *et al.* A multiparametric magnetic resonance imaging-based risk model to determine the risk of significant prostate cancer prior to biopsy. *BJU International* **120**, 774-781 (2017). https://doi.org/10.1111/bju.13814

13 Kinnaird, A. *et al.* A prostate cancer risk calculator: Use of clinical and magnetic resonance imaging data to predict biopsy outcome in North American men. *Canadian Urological Association journal = Journal de l'Association des urologues du Canada* **16**, E161-e166 (2022). https://doi.org/10.5489/cuaj.7380

14 Hyndman, M. E. *et al.* Development of an effective predictive screening tool for prostate cancer using the ClarityDX machine learning platform. *npj Digital Medicine* **7**, 163 (2024). https://doi.org/10.1038/s41746-024-01167-9
